# Prevalence of Severe Depression in Iranian Women with Breast Cancer: A Meta-Analysis

**DOI:** 10.1155/2020/5871402

**Published:** 2020-05-12

**Authors:** Parvaneh Isfahani, Marziye Arefy, Monire Shamsaii

**Affiliations:** Department of Healthcare Services Management, School of Public Health, Zabol University of Medical Sciences, Zabol, Iran

## Abstract

**Background:**

Breast cancer is one of the most common cancers in women and has mental and emotional effects, which lead to a decline in their quality of life. This study is aimed at determining the prevalence of severe depression in Iranian women with breast cancer.

**Methods:**

This meta-analysis study was conducted at Zabol University of Medical Sciences in 2019. Seven electronic databases were searched and evaluated for original research papers published on the prevalence of severe depression in Iranian women with breast cancer. Finally, 13 articles were selected and analyzed via Comprehensive Meta-Analysis software.

**Results:**

Overall, the prevalence of severe depression in Iranian women with breast cancer was 11% (95% CI (7.2, 16.5)). The highest prevalence was 44% in Tehran City in 2015 (95% CI (31, 57.9)), and the lowest prevalence was 0.8% in Qom City in 2017 (95% CI (0.01, 6.2)). A significant statistical correlation was observed between the prevalence of severe depression and sample size (*p* < 0.05).

**Conclusion:**

According to the results of this study, the prevalence of severe depression affects more than one-tenth of women with breast cancer.

## 1. Introduction

Breast cancer is the most common cancer in developed countries among women. According to the World Health Organization (WHO), breast cancer represents 10% of all cancers diagnosed worldwide annually [1]. About 2.1 million women worldwide are diagnosed with breast cancer annually. This type of cancer is the second major cause of cancer death in women after lung cancer. In 2018, it was estimated that 627,000 women died from breast cancer, which was 15% of all cancer deaths among women. Although breast cancer rates are higher among women in more developed countries, its rate is increasing in nearly every region globally [2]. In Iran, breast cancer occurs at least a decade earlier than in developed countries. According to 2016 statistics, 1 in 10 to 15 Iranian women are likely to be diagnosed with breast cancer [3, 4]. By 2016, almost 40,000 Iranian women had been diagnosed with breast cancer, and this number was expected to increase annually by about 7,000 [5].

Breast cancer in women is associated with more severe psychological and emotional effects than other types of cancer. Despite the widespread advances in the detection and treatment of breast cancer, the resulting pain, fear of death, reduced functional capacity, adverse effects of treatment, and lack of financial and social support have increased the propensity for mental disorders in these cancer patients [6]. Many women develop depression over the course of cancer detection and treatment. Depression is a common comorbidity of cancer that has a detrimental effect on the quality of life, treatment adherence, and potential survival. Cancer patients are particularly susceptible to contemplate suicide and self-harm. The prevalence of depression in women with breast cancer varies between 1.5% and 50% [7, 8]. For example, a 2017 study reported that the prevalence of depression was 22% in women with breast cancer in an Indian city [8]. Also, another study in China (2009) indicated that 26% of women with breast cancer suffered from depression symptoms [9].

The emotional response of women to breast cancer ranges from mild mood changes to severe anxiety and depression. Women diagnosed with breast cancer need time to adjust and return to “normal.” However, about 20% to 30% of breast cancer patients experience anxiety, depression, impaired functioning, and low self-esteem following diagnosis. Given the time and method of examination, these effects can persist for a long period of time after detection and treatment and are more common in younger women [10].

Depression reduces the quality of life of breast cancer patients in all fields except family functioning. Treatment of depression in women with breast cancer improves their quality of life and may extend their lifespan [11–13].

In this regard, there are several studies on the prevalence of depression among breast cancer patients in various regions of Iran [3, 11–14]. For example, a study in Sanandaj City in 2017 showed that about 50% of breast cancer patients had mild to severe depression [3]. In this regard, another study on depression in Kermanshah City (2009) reported a prevalence rate of 42.3% [10]. Moreover, in a 2011 study in Isfahan City, the prevalence rate of depression was reported to be 34.5% [6]. However, these studies cannot provide a comprehensive view of this problem for the entire country. Therefore, integrating the results of validated studies in this area can provide better recommendations for policymakers, enabling them to make evidence-based policies. The purpose of this research was to conduct a meta-analysis of the prevalence of severe depression in Iranian women with breast cancer.

## 2. Methods

The present research is a meta-analysis conducted in 2019 at Zabol University of Medical Sciences. It followed the preferred reporting items for systematic review and meta-analysis (PRISMA) guideline [15]. This meta-analysis was independently carried out by searching international and domestic databases (PubMed through MEDLINE, Web of Science, Scopus, Science Direct, PsycINFO, Magiran, and Scientific Information Databases (SID)) for literature published until December 2019. In addition, the Google Scholar search engine was used to access articles focusing on the topic of severe depression in Iranian women with breast cancer.

Search terms included breast neoplasms, cancer, tumor, women, depression, depression disorders, and Iran by using the AND and OR operators. Also, the manual search and reference lists of identified articles were used to find more relevant articles. The initial search was carried out by MSH and MA. Then, an additional search was conducted by PI. The data were extracted and evaluated by PI, MSH, and MA. Finally, the final analysis was done by PI.

The search was initially done in March 2019 and then updated in December 2019, not finding any additional studies that met the inclusion criteria. Only cross-sectional studies on the prevalence of severe depression in women with breast cancer in Iran were included. Moreover, exclusion criteria included articles in any language other than English and Persian, articles published after December 2019, incomplete texts, redundant studies, and dissertations (lack of access). In additional screening, articles in which the full text did not mention severe depression average were excluded (Table 1).

**Figure 1:**
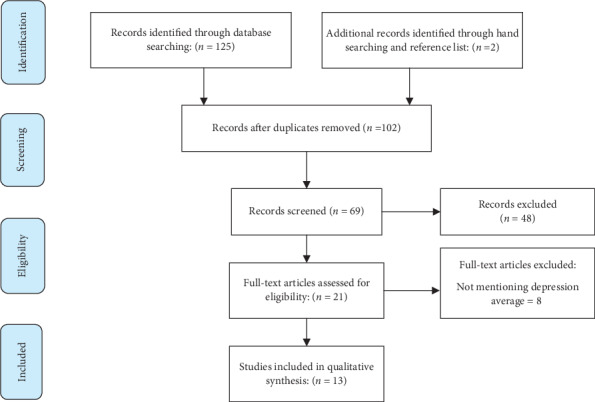
Flowchart of selection and review of articles based on the PRISMA statement.

The initial search resulted in 127 articles. After excluding duplicates and irrelevant articles, 102 studies were selected for abstract examination. After reviewing the abstracts, 48 articles were removed. Also, 8 articles were removed after examining the full texts. Finally, 13 studies were found eligible for inclusion in this systematic review and meta-analysis.  Figure 1 demonstrates the search process.

The quality of the 13 included articles was assessed independently by two authors (MA and MSH) by using the 15-point instrument of Mitton et al. [16] (see the appendix). Each item was given a score of 0 (not present or reported), 1 (present but low quality), 2 (present and midrange quality), or 3 (present and high quality). Criteria for the quality assessment included literature review and identification of research gaps; research questions, hypotheses, and design; population and sampling; data collection process and instruments; and analysis and reporting of results. Disagreements were resolved through discussion or by consulting a third reviewer (PI) if necessary. Finally, moderate- and high-quality studies were only included in this review and meta-analysis. Cohen's kappa coefficient was 0.61 (*p* = 0.004).

Data were extracted from each study based on the title of the article, the name of the first author, year done, average of age, sample size, tool, statistical society, methodology, location of study, and intensive depression prevalence, and an Excel spreadsheet was used for data entry.

Data were analyzed via the Comprehensive Meta-Analysis software (Version 2.2.064). Cochran's *Q* test and *I*^2^ index were used to test heterogeneity. The *I*^2^ index was 88.31%, indicating the heterogeneity of the studies (*I*^2^ values below 25%, between 25% and 75%, and above 75% indicate low, medium, and high heterogeneity, respectively). Therefore, a random-effects model was used in this meta-analysis. Publication bias was examined by using Egger's test, and a *p* value of 0.051 was obtained, indicating that publication bias was not statistically significant.

Finally, by using the metaregression function, the effect of variables, which potentially accounted for the heterogeneity in the included studies, was examined. The point estimate of the prevalence of depression was calculated at the 95% confidence interval (CI) in forest plots, where the size of the box indicates the weight of each study, and the horizontal line indicates the 95% CIs.

## 3. Results

Overall, 13 articles were identified (Table 2). Most of the studies were done in the years 2001, 2012, 2015, and 2017. Most of the studies were conducted in Tehran, Kermanshah, and Kerman cities.

To identify the original authors and articles, the number of citations was examined by Google Scholar. Articles by Vahidinia (260), Nikbakhsh (79), and Montazeri (65) had the highest number of citations [3] (Table 3).

**Figure 2:**
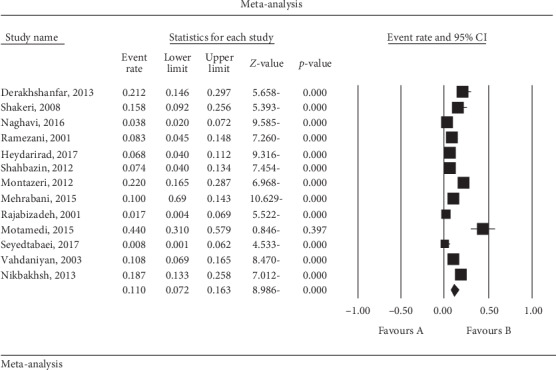
Forest plot of the included studies based on the random-effects model.

Based on the random-effects model, the overall prevalence rate of severe depression was 11% (95% CI (7.2, 16.5)). The lowest prevalence was observed in Qom City in 2017 at 0.8% (95% CI (0.01, 6.2)), and the highest prevalence was observed in Tehran in 2015 at 44% (95% CI (31, 57.9)) (Figure 2).

The results were summarized by the sample size, article quality, type of instrument, and geographic region (Table 4). In this study, severe depression in women with breast cancer was more prevalent in the northern region of Iran than in other regions. Most of the studies used the Zung Self-Rating Depression Scale (SDS) to measure depression. Moreover, studies with sample sizes smaller than 200 reported higher prevalence rates. Finally, studies with higher quality reported higher prevalence rates than those with medium quality.

The results of the evaluated heterogeneity indicated a high level of heterogeneity among the included studies (*Q* = 104.41; *p* = 0.0001). Thus, the variables were entered into the metaregression model to identify those that potentially caused heterogeneity.  Table 5 indicates that the sample size contributed to the heterogeneity of studies on the prevalence of severe depression in women with breast cancer.

## 4. Discussion

Based on the random-effects model, the prevalence of severe depression in Iranian women with breast cancer was found to be 11%. Several studies have examined the prevalence of depression in breast cancer patients worldwide. For example, in an Indian city (2017), the prevalence of depression was reported to be 22% [7], and this prevalence was the same as in a Malaysian city in 2015 [26]. Moreover, a 2013 systematic review reported a prevalence rate of 1% to 56% by surveying 32 eligible studies [8]. The prevalence of severe depression in Iran was reported to be lower than in other countries regarding breast cancer. This could be attributed to the limited number of studies conducted in Iran and small sample sizes used in most studies. However, the special conditions of this disease and treatment process trigger complex mechanisms and worsen a wide range of psychological problems in these patients.

A small number of systematic and meta-analysis studies have examined the prevalence of depression in Iranian women with breast cancer and reported different results [5, 27, 28]. Differences in the quality of review studies and their inclusion and exclusion criteria lead to different results. In these review studies, different aspects of the prevalence of depression in Iranian women with breast cancer were considered. For example, Jafari et al. by reviewing articles published in journals and papers presented in congresses found 8 articles related to the prevalence of depression in Iranian women with breast cancer in the period 2001-2016 [5]. In this study, the prevalence of depression was not reported, and it was only stated that most of the studies reported a mild prevalence of depression in Iranian women with breast cancer. Saeedi et al. reviewed 56 related articles during 1991-2017. In this study, the psychological consequences of breast cancer in Iran in seven classes (anxiety, body image, coping strategies, depression, fatigue, quality of life, and sexual function) were examined and the effect size of these outcomes was assessed [27]. Gharaei et al. examined 18 articles related to the prevalence of depression in Iranian women with breast cancer in 2000-2018. The prevalence in this study was mentioned about 46.83% [28]. However, this study did not determine which levels of depression (mild, moderate, and severe) were assessed. In the present study, the prevalence of severe depression in Iranian women with breast cancer has been considered.

The present research showed that with a unit increase in the sample size, the prevalence of severe depression in Iranian women with breast cancer decreases by 0.006. In other words, studies that use a small sample unintentionally create a sampling bias and consequently cannot provide valuable information for health policymakers and hospital managers. Therefore, studies on the prevalence of depression in breast cancer patients must ensure a representative sample and use of appropriate sampling techniques.

The prevalence of severe depression among women with breast cancer was higher in the northern region of Iran than in other regions of the country. Given that only one study was conducted on this population, this finding must be interpreted with caution. Furthermore, the results of a study on breast cancer incidence rate in Golestan Province reported an incidence rate of 28% between 2004 and 2009 [29]. One of the reasons for the high incidence rate of this disease is the air pollution and stress of living in some of the cities in these regions. On the other hand, modernization of the country, which is more prominent in larger cities, not only has changed dietary habits but also has led to cultural shifts, such as the increase in the age of marriage and age at first birth, which are common risk factors for breast cancer [30].

Being diagnosed with breast cancer can cause depression in patients. Since depression is a key risk factor in the survival of cancer patients and important factor in the treatment of patients, the timely detection and treatment can significantly help the treatment and rehabilitation of these patients and improve their social functioning. On the other hand, studies on the prevalence of severe depression have only been conducted in a limited number of Iran's provinces and are not comprehensive in terms of coverage and generalizability.

The prevalence of severe depression in women with breast cancer varied depending on the type of instrument used. Therefore, the differences observed in the results of the included studies could be partly attributed to the instrumentation.

The main cause of depression in women with breast cancer is metastasis-induced pain and reduced physical and social functioning. Social support can play an important role in the health and well-being of these patients [31, 32]. Hopeful thoughts allow breast cancer patients to care more about their disease and take part in the necessary interventions. Studies have shown that social support and participation in social activities can have a direct impact on the health of these patients [33, 34].

Like many other articles, this study also has some limitations as follows: (a) there is a wide variety of tools used for collecting data, (b) these studies are carried out in a small number of cities in Iran, and (c) there was the lack of valuable information for a detailed survey. It is recommended that future researchers should conduct quantitative studies in the other provinces of Iran. In addition, further qualitative research is recommended to complement quantitative studies and provide a more comprehensive picture of the prevalence of severe depression among breast cancer patients in Iran.

Despite the challenges this review faces in analyzing literature, the results of this study could be valuable in identifying areas of depression in breast cancer which need improvement. This information could be useful for informing health system policymakers, healthcare professionals, and patients alike, leading to a raised awareness which could have many beneficial outcomes, including reducing depression in cancer patients.

## 5. Conclusion

The present research used meta-analysis to determine the prevalence of severe depression in Iranian women with breast cancer and provided useful information for policymakers and administrators in Iran's health sector. The prevalence of severe depression affects more than one-tenth of women with breast cancer. However, because of the limited number of studies and small sample sizes, the present findings must be interpreted with caution. On the other hand, since mental health has a significant role in the survival and recovery of cancer patients, the findings of this study are recommended to be used in cancer treatment centers to provide psychological support to cancer patients.

## Figures and Tables

**Table 1:** Search stages.

Databases	Search strategy	Preliminary searches	Piloting of the study selection process^∗^	Formal screening of search results against eligibility criteria^∗∗^
PubMed	((cancer OR tumor) AND (breast neoplasm) AND (depression OR depressive disorders) AND (women) AND (Iran))	14	7	4
Scopus	ALL(cancer) AND TITLE-ABS-KEY(“breast neoplasm”) AND TITLE-ABS-KEY (depression) TITLE-ABS-KEY(women) AND TITLE-ABS-KEY (Iran)	10	5	4
Web of Science	(ALL=(cancer OR tumor) AND ALL=(breast neoplasm) AND ALL=(depression OR depressive disorders) AND ALL=)women AND ALL= Iran))	4	3	1
Science Direct	(cancer) AND (“breast neoplasms”) AND (depression) AND women AND Iran	9	7	2
PsycINFO	“(cancer) AND (breast) AND (depression) AND (women) AND (Iran)”	1	1	0
Google Scholar	“Breast Neoplasms” AND “depression” AND “Cancer” AND “Women” AND “Iran”	27	19	7
SID	(cancer) AND (breast) AND (depression) AND (women) AND (Iran)	34	25	5
Magiran	(cancer) AND (breast) AND (depression) AND (women) AND (Iran)	26	15	7

Note: ^∗^exclusion based on relevancy to present study; ^∗∗^ exclusion based on duplicate and study criteria.

**Table 2:** Characteristics of the included studies.

Number	Author	Year	Sample	Place	Prevalence	Tool	Age average	Quality article	Reference
1	Derakhshanfar	2013	111	Hamedan	23/5	Beck questionnaire	11/68 ± 47/05	14	[4]
2	Shakeri	2008	78	Kermanshah	12/3	Zung questionnaire	45/15	12	[10]
3	Naghavi	2016	240	Esfahan	9/2	Beck questionnaire	46	14	[11]
4	Ramezani	2001	120	Kerman	10	Beck questionnaire	47/53	15	[17]
5	Heydarirad	2017	200	Sanandaje	13/5	Beck questionnaire	—	12	[3]
6	Shahbazin	2012	127	Kermanshah	9/4	DASS questionnaire	48	11	[18]
7	Montazeri	2012	177	Tehran	39	HADS questionnaire	49/5	12	[19]
8	Nikbakhsh	2013	150	Babol	28/1	HADS questionnaire	—	14	[20]
9	Mehrabani	2016	260	Neyshaboor	26	DASS questionnaire	14/55 ± 55/91	15	[21]
10	Rajabizadeh	2001	110	Kerman	1/9	Beck questionnaire	16/2 ± 50/6	10	[22]
11	Motamedi	2015	50	Tehran	22	Beck questionnaire	46/94	13	[23]
12	Seyedtabaei	2017	109	Ghom	0/9	R-90-SCL questionnaire	—	11	[24]
13	Vahdaniyan	2003	167	Tehran	18	HADS questionnaire	47/2 ± 13/5	13	[25]

**Table 3:** Number of citations of Google Scholar.

First author	Google Scholar
Derakhshanfar	10
Shakeri	5
Naghavi	35
Ramezani	0
Heidarrad	5
Shahbaziyan	2
Montazeri	65
Nikbakhsh	79
Mehrabani	1
Rajabirad	7
Mohtamedi	0
Seyedtabai	5
Vahdani niya	260

**Table 4:** Subgroup analyses of the included studies.

Variable	No. of studies	Prevalence 95% CI	*I* ^2^	*P*
Sample size				
<200	10	13.5% (8.7-20.4)	86.35	*p* ≤ 0.001
>200	2	6.4% (2.5-15.8)	85.17	*p* ≤ 0.001
=200	1	6.8% (4.0-11.2)	—	—
Regional				
Center	5	11/2% (4/2-26/4)	93.62	*p* ≤ 0.001
East	3	7.0% (3.6-13.3)	66.14	*p* ≤ 0.001
North	1	18.7% (13.3-25.8)	—	*p* ≤ 0.001
West	4	12.4% (6.6-22.2)	84.41	*p* ≤ 0.001
Tool				
Beck	6	9.7% (3.9-22.3)	93.16	*p* ≤ 0.001
DASS	2	9.2% (6.7-12.7)	0.000	*p* ≤ 0.001
HADS	3	16.9% (11.3-24.6)	74.07	*p* ≤ 0.001
Zung	1	19.6% (12.2-19.9)	—	*p* ≤ 0.001
R-90-SCL	1	0.8% (0.01-6.2)	—	*p* ≤ 0.001
Article quality				
High	6	12.2% (6.7-4.37)	84.96	*p* ≤ 0.001
Moderate	7	9.5% (4.4-19.2)	91.45	*p* ≤ 0.001

**Table 5:** Results of metaregression.

Variable	Article number	Coefficient	SE	PV
Year	13	0/01	0/016	0/29
Sample	13	-0/006	0/001	0/0001
Age average	13	0/0007	0/003	0/82

## Appendix

Quality Rating Sheet

Ref ID: ________

KTE Empirical Article Quality Rating Sheet

0 – not present or reported anywhere in the article

1 – present but low quality

2 – present and midrange quality

3 – present and high quality

________ 1. Literature Review: Directly related recent literature is reviewed and research gap(s) identified.

________ 2. Research Questions and Design: A priori research questions are stated, and hypotheses, a research purpose statement, and/or a general line of inquiry is outlined. A study design or research approach is articulated.

________ 3. Population and Sampling: The setting, target population, participants, and approach to sampling are outlined in detail.

________ 4. Data Collection and Capture: Key concepts/measures/variables are defined. A systematic approach to data collection is reported. Response or participation rate and/or completeness of information capture is reported.

________ 5. Analysis and Results Reporting: An approach to analysis and a plan to carry out that analysis is specified. Results are clear and comprehensive. Conclusions follow logically from findings.

________ /15 = Total Score

## References

[1] World Health Orgniztion (2016). *Guidelines for the early detection and screening of breast cancer*.

[2] WHO (2019). *Breast cancer*.

[3] Heidarirad F., Yarahmadi M., Heidarirad H., Shafeie M. (2018). Evaluation of prevalence of depression and its related factors among women with breast cancer referred to the Radiotherapy Center of Tawhid Hospital of Sanandaj, Iran in 2017. *Scientific Journal of Nursing, Midwifery and Paramedical Faculty*.

[4] Derakhshanfar A., Niayesh A., Abbasi M., Ghalaeeha A., Shojaee M. (2013). Frequency of depression in breast cancer patients: a study in Farshchian and Besat Hospitals of Hamedan during 2007-8.. *Iranian Journal of Surgery*.

[5] Jafari A., Goudarzian A. H., Nesami M. B. (2018). Depression in women with breast cancer: a systematic review of cross-sectional studies in Iran. *Asian Pacific Journal of Cancer Prevention*.

[6] Musarezaie A., Momeni-Ghaleghasemi T., Gorji M. (2014). Survey the anxiety and depression among breast cancer patients referred to the Specialized Isfahan Hospital of Cancer. *Journal of Health System Research*.

[7] Purkayastha D., Venkateswaran C., Nayar K., Unnikrishnan U. G. (2017). Prevalence of depression in breast cancer patients and its association with their quality of life: a cross-sectional observational study. *Indian Journal of Palliative Care*.

[8] Zainal N. Z., Nik-Jaafar N. R., Baharudin A., Sabki Z. A., Ng C. G. (2013). Prevalence of depression in breast cancer survivors: a systematic review of observational studies. *Asian Pacific Journal of Cancer Prevention*.

[9] Chen X., Zheng Y., Zheng W. (2009). Prevalence of depression and its related factors among Chinese women with breast cancer. *Acta Oncologica*.

[10] Shakeri J., Golshani S., Jalilian E. (2009). Frequency of depression in patients with breast cancer referring to chemotherapy centers of Kermanshah educational centers (2007-2008). *Asian Pacific Journal of Cancer Prevention*.

[11] Tomich P. L., Helgeson V. S. (2002). Five years later: a cross-sectional comparison of breast cancer survivors with healthy women. *Psychooncology*.

[12] Mols F., Vingerhoets A. J., Coebergh J. W., van de Poll-Franse L. V. (2005). Quality of life among long-term breast cancer survivors: a systematic review. *European Journal of Cancer*.

[13] Yen J.-Y., Ko C. H., Yen C. F. (2006). Quality of life, depression, and stress in breast cancer women outpatients receiving active therapy in Taiwan. *Psychiatry and Clinical Neurosciences*.

[14] Taghavi M., Kalafi E., Talei A., Dehbozorgi G., Taghavi S. M. (2011). Investigating the relation of depression and religious coping and social support in women with breast cancer. *Journal of Isfahan Medical School*.

[15] Moher D., Liberati A., Tetzlaff J., Altman D. G., The PRISMA Group (2009). Preferred reporting items for systematic reviews and meta-analyses: the PRISMA statement. *PLoS Medicine*.

[16] Mitton C., Adair C. E., Mckenzie E., Patten S. B., Perry B. W. (2007). Knowledge transfer and exchange: review and synthesis of the literature. *Milbank Quarterly*.

[17] Ramezani T. (2001). The rate of depression and needs counseling in women with breast cancer chemotherapy centers in Kerman. *Iranian Journal of Psychiatry and Clinical Psychology*.

[18] Shahbazin S., Mousavi S. A., Khaledi Paveh B., Nasury M., Aazami S. (2014). Relationship between psychological status and coping strategies in women with breast cancer in Kermanshah, 2012. *Journal of Clinical Research in Paramedical Sciences*.

[19] Montazeri A., Sajadian A., Ebrahimi M., Akbari M. E. (2005). Depression and the use of complementary medicine among breast cancer patients. *Supportive Care in Cancer*.

[20] Nikbakhsh N., Moudi S., Abbasian S., Khafri S. (2014). Prevalence of depression and anxiety among cancer patients. *Caspian Journal of Internal Medicine*.

[21] Fatemeh M., Farzaneh B., Elaheh R. T., Mehdi B., Bahare G. C. (2016). Unpleasant emotions (stress, anxiety and depression) and it is relationship with parental bonding and disease and demographic characteristics in patients with breast cancer. *Iranian Journal of Breast Diseases*.

[22] Rajabizadeh G. A. (2005). Determination of factors related to depression in cancer patients of the oncology ward in Kerman. *Journal of Kerman University of Medical Sciences*.

[23] Motamedi A., Haghighat S., Khalili N. (2015). The correlation between treatment of depression and quality of life in breast cancer survivors. *Iranian Journal of Breast Diseases*.

[24] Seyyed Tabaei S. R., Rahmatinejad P., Sehat R. (2015). The prevalence of behavioral symptoms of psychological disorders in cancer patients. *Journal of Thought & Behavior in Clinical Psychology*.

[25] Vahdaninia M., Omidvari S., Montazeri A. (2010). What do predict anxiety and depression in breast cancer patients? A follow-up study. *Social Psychiatry and Psychiatric Epidemiology.*.

[26] Hassan M. R., Shah S. A., Ghazi H. F., Mohd Mujar N. M., Samsuri M. F., Baharom N. (2015). Anxiety and depression among breast cancer patients in an urban setting in Malaysia. *Asian Pacific Journal of Cancer Prevention*.

[27] Saeedi N. R., Sharbaf H. A., Ebrahimabad M. J., Kareshki H. (2019). Psychological consequences of breast cancer in Iran: a meta-analysis. *Iranian Journal of Public Health*.

[28] Gharaei H. A., Dianatinasab M., Kouhestani S. M. (2019). Meta-analysis of the prevalence of depression among breast cancer survivors in Iran: an urgent need for community supportive care programs. *Epidemiology and Health*.

[29] Taheri N. S., Nosrat S. B., Aarabi M. (2012). Epidemiological pattern of breast cancer in Iranian women: is there an ethnic disparity. *Asian Pacific Journal of Cancer Prevention*.

[30] Tfaily M. A., Naamani D., Kassir A. (2019). Awareness of colorectal cancer and attitudes towards its screening guidelines in Lebanon. *Annals of Global Health*.

[31] Ataollahi M., Masoumi S. Z., Shayan A., Roshanaei G., Sedighi S. (2016). Comparison of perceived social support and perceived stress in women with and without breast cancer referred to Mahdieh MRI Center of Hamedan in 2013. *Pajouhan Scientific Journal*.

[32] Kodzi I. A., Gyimah S. O., Emina J., Ezeh A. C. (2011). Religious involvement, social engagement, and subjective health status of older residents of informal neighborhoods of Nairobi. *Journal of Urban Health*.

[33] Kamen C., Cosgrove V., McKellar J., Cronkite R., Moos R. (2011). Family support and depressive symptoms: a 23-year follow-up. *Journal of Clinical Psychology*.

[34] Maly R. C., Umezawa Y., Leake B., Silliman R. A. (2005). Mental health outcomes in older women with breast cancer: impact of perceived family support and adjustment. *Psycho-Oncology*.

